# Body Composition Changes in Adolescents Who Underwent Bariatric Surgery: A Systematic Review and Meta-analysis

**DOI:** 10.1007/s13679-023-00549-6

**Published:** 2024-01-03

**Authors:** Andréa Bezerra, Giorjines Boppre, Laura Freitas, Francesca Battista, Federica Duregon, Sara Faggian, Luca Busetto, Andrea Ermolao, Hélder Fonseca

**Affiliations:** 1 Research Centre in Physical Activity, Health and Leisure (CIAFEL), Faculty of Sport, University of Porto, Porto, Portugal; 2 Laboratory for Integrative and Translational Research in Population Health (ITR), Porto, Portugal; 3 Human Motricity Research Center, University Adventista, Chillean, Chile; 4 Sports and Exercise Medicine Division, Department of Medicine, University of Padova, Padova, Italy; 5 Department of Medicine, University of Padova, Padova, Italy; 6 Clinical Network of Sports and Exercise Medicine of the Veneto Region, Padova, Italy

**Keywords:** Bariatric surgery, Body composition, Fat mass, Lean mass, Adolescents, Pediatrics

## Abstract

**Purpose of Review:**

The purpose of this review and meta-analysis is to characterize the changes in body composition of children and adolescents who underwent bariatric surgery and identify possible negative effects of performing this procedure during pediatric ages.

**Recent Findings:**

Bariatric surgery in children and adolescents is an emerging strategy to promote higher and faster body weight and fat mass losses. However, possible negative effects usually observed in surgical patients’ muscle-skeletal system raise a major concern perform this intervention during growth. Despite these possible issues, most experimental studies and reviews analyze bariatric surgery’s effectiveness only by assessing anthropometric outcomes such as body weight and BMI, disregarding the short- and long-term impact of bariatric surgery on all body composition outcomes.

**Summary:**

Bariatric surgery is effective to reduce fat mass in adolescents, as well as body weight, waist circumference, and BMI. Significant reduction in lean mass and fat-free mass is also observed. Bone mass seems not to be impaired. All outcomes reduction were observed only in the first 12 months after surgery. Sensitivity analysis suggests possible sex and type of surgery-related differences, favoring a higher fat mass, body weight, and BMI losses in boys and in patients who underwent RYGB.

**Supplementary Information:**

The online version contains supplementary material available at 10.1007/s13679-023-00549-6.

## Introduction

The prevalence of obesity is growing worldwide [1]. This disease is a significant public health issue [2] as it favors the development of several other comorbidities such as diabetes [3], cardiovascular disease [4], and several types of cancer [5] compromising quality of life and mortality risk. Obesity is particularly concerning during childhood and adolescence because these developmental phases are pivotal for the acquisition of healthy lifestyle habits [6] and due to the associated consequences of the early instalment of overweight and obesity for adult cardiometabolic disease risk [7••]. In this context, severe cases of childhood and adolescent obesity are particularly worrisome [8].

Bariatric surgery (BS) is a well-established treatment for obesity [9, 10] and many of its related comorbidities. This evidence is well documented in adults [11] and also, increasingly, in adolescents [12, 13]. However, the greater reduction in body weight promoted by BS is also tied to substantial changes in other body composition components [14] such as significant decreases in muscle [15•] and bone [16, 17•] mass. Consequently, when performed in adolescents, BS-induced lean mass losses raise some concerns due to their possible negative influence during the growth and development phase [18, 19••]. The energy deprivation and accelerated weight loss in the first months post-BS, as well as the risk of nutritional deficiencies [20], which are more prevalent after malabsorptive procedures such as the Roux-en-Y gastric bypass (RYGB) and which are aggravated by the low adherence of adolescents to nutritional supplementation recommendations [21] could have detrimental metabolic and musculoskeletal consequences, especially during adolescents growth [22]. Despite these concerns, and also considering the new expanded definition of adolescence, which goes now from 10 to 24 years of age [23], available follow-up data in children and adolescents who underwent BS suggest a normal growth at 2 [24] and 5 years [25] after sleeve gastrectomy (SG). Nevertheless, the long-term potential consequences of BS in pediatric ages remain controversial and might differ substantially according to the specific population analyzed [26] and bariatric procedure employed [27].

Despite the increasing adoption of bariatric procedures for adolescents with severe obesity, only few studies with a small number of subjects have assessed changes in body composition in this population. In addition, there is a lack of systematic reviews with meta-analysis analyzing the available data on the effects of BS on adolescent’s body composition. Consequently, the aim of this systematic review with meta-analysis is to characterize the short- and long-term effects of BS on body composition of children, adolescents, and young adults and to determine how these changes are influenced by different bariatric procedures.

## Methods

### Design

This systematic review followed PRISMA guidelines with PROSPERO registration number CRD42022363749.

### Eligibility Criteria

This systematic review included longitudinal observational studies and randomized or non-randomized controlled trials in the English language, carried out with children and adolescents (until 24 years) [23] with obesity (BMI ≥ 35 kg.m^−2^), who underwent BS and whose body composition had been assessed before and after the bariatric surgery procedure. There were no limitations regarding the type of instruments used for assessing body composition or patients’ obesity-related comorbidities. Exclusion criteria were (i) adult patients over 24 years of age; (ii) absence of body composition data before and after BS; and (iii) cross-sectional studies, reviews, commentaries, perspective studies, and editorials. All studies that met the inclusion criteria were considered for analysis.

### Search Strategy

The systematic search was conducted in October 2022 in four databases: Pubmed/MEDLINE^®^, EBSCO^®^, Web of Science^®^, and Scopus^®^. The search terms used for this review were ((“Bariatric Surgeries” OR “Bariatric Surgery” OR “Metabolic Surgery” OR “Metabolic Surgeries” OR “Bariatric Surgical Procedures” OR “Bariatric Surgical Procedure” OR “sleeve gastrectomy” OR “Roux-en-Y gastric bypass” OR “RYGB” OR “gastric bypass”) AND (Bone OR “Bone mineral density” OR “bone density” OR BMD OR “bone mineral content” OR “bone content” OR BMC OR “fat mass” OR “lean mass” OR “Body fat mass” OR “fat-free mass” OR “body lean mass” OR “body composition”)) AND (Child OR children OR childhood OR Pediatric OR adolescent OR adolescents OR adolescence OR teen OR teens OR teenager OR teenagers OR youth OR young OR “Pediatric obesity” OR “child obesity” OR “childhood obesity” OR “adolescent obesity” OR “infant obesity” OR “infantile obesity”).

### Study Selection

**Fig. 1:**
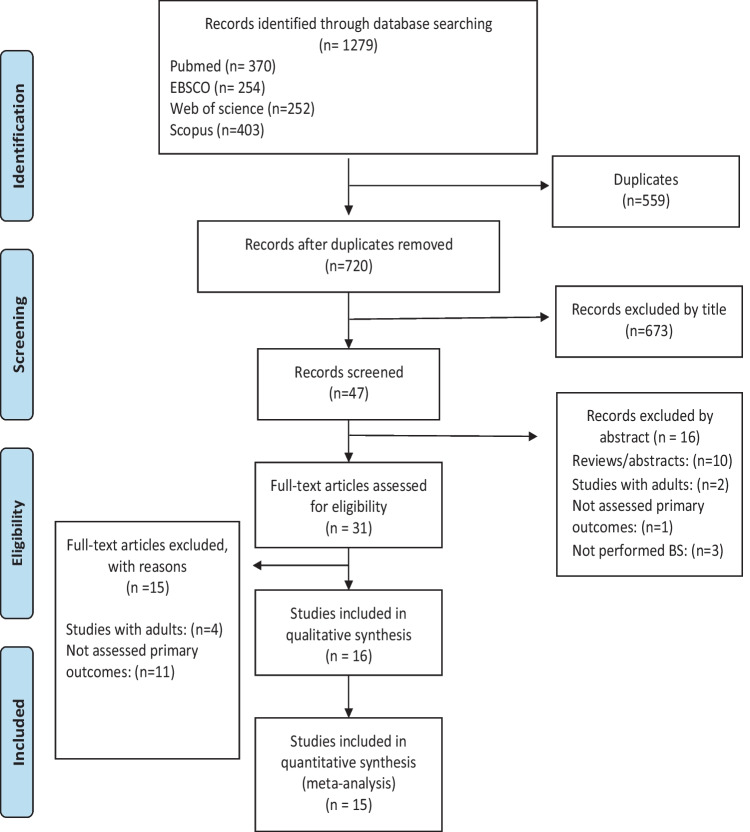
Studies search and selection process

Articles were recorded to an Endnote database (Endnote X9, Thomson Reuters, San Francisco, California). Duplicates were removed and the remaining articles were screened by title, abstract, and, finally, full text by two independent authors (A.B. and L.F.). Disagreements were solved by discussion between the two reviewers. The selection procedures are exposed in Fig. 1. Selected articles had to obey the PECOS strategy (Table 1).

**Table 1:** PECOS strategy used for study selection

Population	Children and adolescents who underwent BS
Exposition	Bariatric surgery procedure
Comparison	Baseline data of the same surgical group
Outcomes	Primary body composition outcomes: fat mass, lean mass, fat-free mass, and bone mass outcomes. Bone mass variables considered were the bone mineral content (BMC) and areal (aBMD) or volumetric bone mineral density (vBMD) at the whole body and lumbar vertebrae
	Secondary anthropometric outcomes: body weight, BMI, and waist circumference
Studies	Randomized or non-randomized clinical trials, or longitudinal observational studies

### Risk of Bias

The Risk of Bias was assessed independently by two authors (A.B. and L.F.) using Risk Of Bias In Non-randomized Studies-of Exposure (ROBINS-E) [28]. This tool consists of seven domains and was considered to assess the quality of observational studies. The quality of evidence for the changes in body composition outcomes after BS was assessed using Grading of Recommendations, Assessment, Development, and Evaluation (GRADE) [29].

### Data Extraction

Authors, publication year, country, study design, sample size, type of surgery, data from baseline, and post-BS assessments (i.e., body weight, body mass index (BMI), waist circumference, lean mass, fat mass, areal and volumetric bone mineral density (aBMD and vBMD, respectively), and bone mineral content (BMC)) were extracted from the articles. When BMC data (mean and standard deviation) were available only in graphics, the authors were contacted to obtain accurate data.

### Data Synthesis

Data from some studies [30, 31, 32••] was converted from standard error [33] and 95% confidence interval [30, 32••] into standard deviation according to the Cochrane Handbook recommendations [34]. Further, when data was provided in the median and interquartile range [35], a conversion was made to mean and standard deviation [36]. Lean mass and fat mass were converted from lb to kg [37]. When data [38] was available only for separated analyses according to sex, mean differences were calculated as being two different studies. This was done for Beamish et al. A (for girls) and Beamish et al. B (for boys). Similarly, the study of Dubnov-Raz et al. [39] was divided into three studies, since they provided data comprising both sexes (Dubnov-Raz et al. A), and data separated by sex, namely Dubnov-Raz et al. B (for girls) and Dubnov-Raz et al. C (for boys). When data about the number of participants [40] or standard deviation were lacking [13], studies were excluded from the meta-analysis.

### Statistical Analysis

A random-effects model was performed for each selected outcome. Pooled effect sizes (ES) were presented as unstandardized mean differences (MD) with a 95% confidence interval (95% CI). First, an overall analysis was performed to explore the bariatric surgery effects in body composition and anthropometric outcomes after 12 months, and, afterward, sub-analyses were conducted separately by sex and bariatric surgery type. A comparison between the outcome’s changes during the first and the second year was also performed. Sensitivity analyses were conducted to detect if any study was responsible for a large proportion of heterogeneity (*I*^2^), which was assessed and qualitatively considered not important if *I*^2^ = 0–40%, moderate if *I*^2^ = 30–60%, substantial if *I*^2^ = 50–90%, and considerable if *I*^2^ = 75–100% [41]. The package “meta” (version 4.11–0) and “metafor” (version 3.8–1) for the R statistical software (version 4.1.0) were used [42]. Overall effects (*z*-value) were considered statistically significant when *p*-value < 0.05.

## Results

### Selection and Identification of Included Studies

From 1279 initial references, 31 studies were selected for full-text analysis, from which 11 [22, 24, 25, 43–50] and four studies [51–54] were excluded after full-text evaluation for not assessing the primary outcomes or for including adults, respectively. Finally, 16 studies matched our inclusion and exclusion criteria and were selected for qualitative analysis, of which 15 were also considered in the meta-analysis (Fig. 1). Among the 16 included studies, ten were performed in the USA [30, 31, 32••, 35, 37, 40, 55, 56, 57, 58••], three in Sweden [38, 59, 60], one in Singapore [13], one in Canada [61], and one in Israel [39] (Table 2).

**Table 2:** General characteristics of the included studies of adolescents who underwent bariatric surgery

Author (Year); Country	Sample Profile	Type of surgery	Instrument/ outcomes assessed	Main findings
	Sample size, sex, age; PS	Follow-up		Changes in body composition
Inge et al. (2007) [55]; USA	n = 5 ♀ age = 18.0 ± 3.0; PS > 5	RYGB: 12 M	DXA FM, LM	↓ BW (p < 0.01); ↓ BMI (p < 0.01); ↓ LM (p = 0.01); ↓ FM (p < 0.01)
Nadler et al. (2009) [37]; USA	n = 36 ♀♂ age = 16.0 ± 1.2; PS = nr	LAGB: 12 M	DXA FM, LM, WB aBMD	↓ BW (p < 0.0001); ↓ BMI (p < 0.0001); ↓ LM (p = 0.0001); ↓FM (p < 0.0001) ↑WB aBMD (p = 0.0004)
Kaulfers et al. (2011) [40]; USA	n = 61(51♀,10♂) age = 17.3 ± 1.9; PS > 4	RYGB: 6, 12, 18, 24 M	DXA WB BMC	↓BW (p < 0.0001); ↓ WB BMC (p < 0.0001)
Butte et al. (2015) [56]; USA	n = 11(8♀,3♂) age = 16.5 ± 0.8; PS > 4	RYGB: 1.5, 6, 12 M	BOD POD FM, FFM	↓ BW (p < 0.0001); ↓ BMI (p < 0.0001); ↓ FFM (p = 0.001); ↓ FM (p < 0.0001)
Dubnov-Raz et al. (2015) [39]; Israel	n = 25(9♀,16♂) age = 16.6 ± 1.5; PS = nr	SG: 12 M	BIA FM, FFM	↓ BW (p < 0.0001); ↓ BMI (p < 0.0001); ↓ FFM (p < 0.001); ↓ FM (p < 0.0001)
Brissman et al. (2017) [59]; Sweden	n = 41(31♀,10♂) age = 14–18; PS > 3	RYGB: 12, 24 M	DXA FM, FFM	↓ BW (p < 0.001); ↓ BMI (p < 0.001); ↓ FFM (p < 0.001); ↓ FM (p < 0.001)
Beamish et al. (2017) [38]; Sweden	n = 72(50♀,22♂); age = 16.5 ± 1.2; PS = nr	RYGB: 12, 24 M	DXA FM, LM, WB BMC/ aBMD	↓ BW (p < 0.001); ↓ BMI (p < 0.001); ↓ LM (p < 0.001); ↓ FM (p < 0.001); ↑BMC (p < 0.001); ↓ aBMD (p < 0.001)
Dargan et al. (2018) [13]; Singapure	n = 13(5♀,8♂); age = 19.1 ± 0.9; PS = nr	SG: 1, 3, 6, 12 M	BIA FM, FFM	↓BW (p < 0.001)
Chu et al. (2019) [61]; Canada	n = 20(15♀,5♂); age = 17.2 ± 0.8; PS > 5	RYGB/SG: 6, 12 M	BIA FM, FFM	↓ BW (p < 0.001); ↓ BMI (p < 0.001); ↓ FFM (p < 0.001); ↓ FM (p < 0.001)
Henfridsson et al. (2019) [60]; Sweden	n = 85(57♀,28♂); age = 16.0 ± 1.2; PS: nr	RYGB: 12, 24 M, 5 Y	DXA LM, FM	↓ BW (p < 0.001); ↓ BMI (p < 0.001); ↓ LM (p < 0.001); ↓ FM (p < 0.001)
Rickard et al. (2019) [31]; USA	n = 12♀ age = 18.8 ± 2.2; PS: nr	SG: 12 M	DXA LM, FM	↓ BW (p < 0.0005); ↓ BMI (p < 0.034); ↓ LM (p < 0.001); ↓ FM (p < 0.001)
Bredella et al. (2020) [57]; USA	n = 26 (19♀, 7♂); age = 18.0 ± 2.1; PS: nr	SG: 12 M	QCT LS vBMD	↓ BW (p < 0.001); ↓ BMI (p < 0.001); ↓ LS vBMD (p = 0.04)
Misra et al. (2020a) [30]; USA	n = 24 (18♀, 6♂). age = 17.8 ± 1.96; PS: nr	SG: 12 M	DXA LM, FM,	↓ BW (p < 0.05); ↓ BMI (p < 0.05); ↓ LM (p < 0.05); ↓ FM (p < 0.05)
Misra et al. (2020b) [32••]; USA	n = 22 (16♀, 6♂). age = 18.3 ± 2.35; PS: nr	SG: 12 M	DXA LM, FM, WB aBMD, LS aBMD	↓ BW (p < 0.05); ↓ BMI (p < 0.05) ↓ LM (p < 0.05); ↓ FM (p < 0.05)
Bredella et al. (2021) [58••]; USA	n = 10 (9♀, 1♂) age = 17.8 ± 2.5; PS: nr	SG: 12 M	QCT LS vBMD	↓ BW (p < 0.0001); ↓ BMI (p < 0.02)
Nimmala et al. (2022) [35]; USA	n = 30 (24♀, 6♂) age = 18.2 ± 0.4; PS: nr	SG: 12 M	DXA LM, FM	↓ BW (p < 0.0001); ↓ BMI (p < 0.0001) ↓ LM (p < 0.0001); ↓ FM (p < 0.0001)

*BIA* bioelectrical impedance analysis, *aBMD* areal bone mineral density, *BMC* bone mineral content, *BMI* body mass index, *BW* body weight, *DXA* dual-energy X-ray absorptiometry, *CSA* cross-sectional area, cross-sectional moment of inertia, *FM* fat mass, *LAPG* laparoscopic adjustable gastric banding, *LM* lean mass, *LS* lumbar spine, *M* months, *nr* not reported, *Pre* preoperative, *PS* pubertal status, *QCT* quantitative computed tomography, *RYGB* Roux-n-y gastric banding, *SG* sleeve gastrectomy, *vBMD* volumetric bone mineral density, *WB* whole body, *Y* years. ♀ female, ♂ male

### General Characteristics of the Population

The whole sample considered in this systematic review comprised 490 adolescents who underwent BS. No studies assessed body composition changes in children under 10 years old. One study did not characterize the subgroup by sex [37], and among the other 15 studies, 329 of the included subjects were girls and 122 boys. Age ranged between 13 and 24 years with a mean age of 17.4 ± 1.6 years. One study reported only that patients were between 14 and 18 years [59]. The pubertal stage was only defined in two studies using the Tanner method [55, 61]. Among the other studies, one only included adolescents above the third Tanner stage [59], whereas two included only patients above the fourth [40, 56] and two above the fifth [55, 61] stage (Table 2).

### Time and Type of Exposure

Adolescents underwent sleeve gastrectomy (SG), RYGB, and laparoscopic adjustable gastric banding (LAGB) in eight [13, 30, 31, 32••, 35, 39, 57, 58••], six [38, 40, 55, 56, 59, 60], and one study [37], respectively. One study included patients from both SG and RYGB [61]. All studies assessed the patients before and after 12 months of surgery. In one study, the baseline assessment was after surgery in three patients (two patients at day 10 and 1 patient at 8-week post-surgery) due to excess body weight which hindered dual-energy X-ray absorptiometry (DXA) assessment [55]. Only one study performed assessments at three time points (2 weeks, the first and third month after BS) [13]. Three studies evaluated patients 6 months post-BS [13, 56, 61] and three studies at 24-month post-BS [38, 40, 60]. The study that performed the longest follow-up, evaluated participants at 5 years after BS [60].

### Primary Outcomes Related to Body Composition

#### Lean Mass, Fat-Free Mass, and Fat Mass

DXA was used to assess body composition in 10 studies. One study assessed lean and fat mass using DXA at the whole body, trunk, and extremities [55]. One study assessed body composition using BOD POD’s air displacement plethysmography [56] and three studies assessed body composition through bioelectrical impedance (BIA) [13, 39, 61]. All these body composition outcomes decreased significantly in the first year after BS in all but one study [13]. The total lean mass lost in the first year after surgery was − 7.98 ± 2.55 kg. After 2 years, an improvement in lean mass could be identified, reducing this loss to − 4.86 ± 1.1 kg. Similarly, fat-free mass losses in the first year were − 7.50 ± 3.16 kg, while in the second-year losses decreased to − 5.92 ± 2.51. Conversely, the mean fat mass loss in the first year was − 29.34 ± 5.60 kg, but it kept decreasing to − 32.56 ± 1.75 kg at the 2-year follow-up.

#### Bone Mass Outcomes

Nine studies analyzed bone mass. DXA was used in six studies, where four assessed aBMD at the whole body [32••, 37, 38] and lumbar spine [32••] and two assessed total body BMC [38, 40]. Two studies also evaluated lumbar spine vBMD through quantitative computed tomography (QCT) [57, 58••]. One study found an increase in aBMD 12 months after surgery [37], whereas another study reported a decrease 24 months after BS [38]. Regarding BMC, one study reported a decrease [40], while another study reported an increase [38] 2 years after surgery. Only one study [57] found a significant decrease in lumbar spine vBMD 12 months after sleeve gastrectomy (*p* = 0.04).

### Secondary Outcomes: Anthropometric Variables

#### Body Weight, BMI, and Waist Circumference

All studies reported weight loss after surgery. The average weight loss in the first year was − 40.48 ± 7.76 kg whereas 2 years after BS mean weight loss was − 44.22 ± 2.8 kg. BMI reduction one and 2 years after surgery was, on average − 13.70 ± 1.86 kg/m^2^ and − 15.53 ± 0.74 kg/m^2^, respectively. Regarding waist circumference, the mean loss in the first year after BS was − 27.6 ± 1.5 cm. No studies assessed this outcome in the second year.

### Study Quality Assessment

According to ROBINS-E, eight studies were classified as having “low risk, except about some concerns” due to aspects present in almost all studies such as differences in follow-up hospital visits, diet, and physical activity counseling [30, 32••, 35, 39, 56, 57, 58••, 61]. Four studies were classified as having “some concerns” especially regarding the use of different DXA scanner models to assess body composition [38, 59], limited information about assessments [31] and lack of information regarding the follow-up protocol after surgery [37]. Three studies were classified as having a “high risk of bias” especially regarding missing data or due to different follow-up intervals [13, 55, 60]. Finally, one study was rated as having a “very high risk of bias” during the ROBINS-E preliminary consideration analysis due to missing data regarding sample size and standard deviation during follow-up and lack of information about assessment timing during the follow-up [40]. Consequently, the analysis of the seven domains for this study was not necessary according to the ROBINS-E tool. In an overall view, for all domains, most studies were rated as having a “low risk of bias.” The second most observed classification in domains related to confounding (D1) and outcome measurements (D6) was “some concerns.” Domain five (D5), related to missing data, was the domain in which studies presented a more frequent “high risk of bias” classification (Supplementary Fig. 1).

### Certainty of Evidence

GRADE analysis revealed that there is a low quality of evidence regarding changes in body composition following BS due to high heterogeneity causing results inconsistency (Supplementary Table 1).

### Meta-analysis

#### Analysis of Body Composition at 12-Month Post-BS

**Fig. 2:**
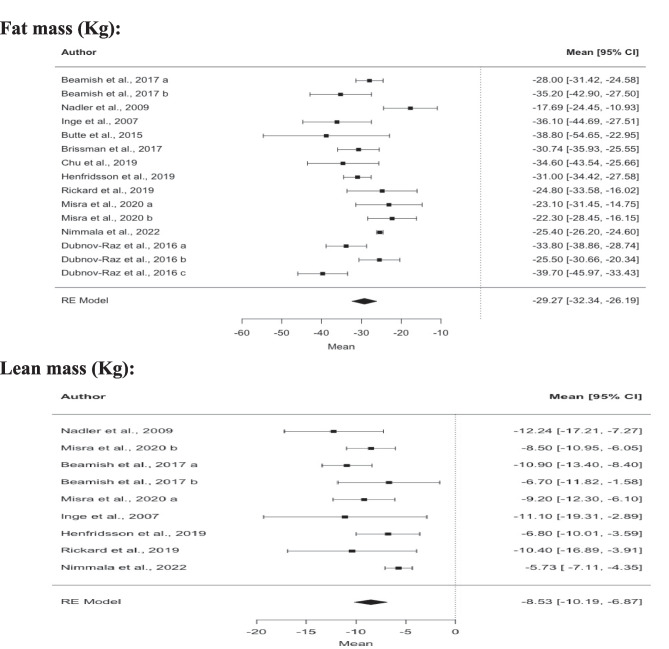

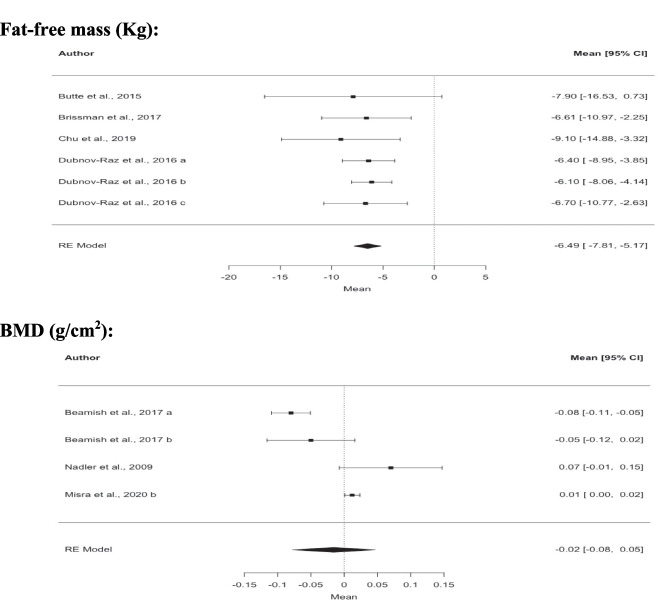
Effects of bariatric surgery on body composition after 12 months. Note: Data are presented as mean difference ± 95% confidence interval. Abbreviations: BMD, bone mineral density; RE model, random effect model

The meta-analysis revealed reductions in all body composition outcomes but bone mass. Fat mass (− 29.3 kg; 95% CI; − 32.3; − 26.2; *z* =  − 18.7; *p* = 0.001), lean mass (− 8.5 kg; 95% CI; − 10.2; − 6.9; *z* =  − 10.1; *p* < 0.001), and fat-free mass (− 6.5 kg; 95% CI − 7.8; − 5.2; *z* =  − 9.6; *p* = 0.001) were all reduced 1 year after surgery (Fig. 2; Table 3). Regarding bone mass outcomes, only whole-body aBMD had enough studies to carry out a meta-analysis. No significant differences were observed in aBMD 1 year after bariatric surgery (− 0.02 g/cm^2^; 95% CI; − 0.08; 0.05; *z* =  − 0.49; *p* = 0.623). Decreases were also observed in our secondary outcomes, namely body weight (− 38.8 kg; 95% CI; − 41.2; − 36.3; *z* =  − 30.8; *p* = 0.001), BMI (− 13.9 kg.mm^2^; 95% CI; − 14.7; − 13.1; *z* =  − 34.5; *p* = 0.001), and waist circumference (− 28.0 cm; 95% CI; − 37.9; − 18.2; *z* =  − 5.6; *p* = 0.001) 1 year after surgery (Table 3).

**Table 3:** Effects of all bariatric surgery procedures

**Outcomes**	**Overall**			
	k	MD (95% CI)	I^2^	Z (p)
**Anthropometrics**				
Body weight (Kg)	18	-38.8 (-41.2; -36.3)	39%	-30.8 (0.001)
BMI (Kg.m^−2^)	18	-13.9 (-14.7; -13.1)	37%	-34.5 (0.001)
Waist circumference (cm)	3	-28.0 (-37.9; -18.2)	73%	-5.6 (0.001)
**Body composition**				
Lean mass (kg)	9	-8.5 (-10.2; -6.9)	56%	-10.1 (< .001)
Free fat mass (kg)	6	-6.5 (-7.8; -5.2)	0%	-9.6 (0.001)
Fat mass (Kg)	15	-29.3 (-32.3; -26.2)	84%	-18.7 (< .001)
BMD (g/cm^2^)	4	-0.02 (-0.08; 0.05)	92%	-0.49 (0.623)
BMD Z-score	4	-0.46 (-1.13; 0.22)	93%	-1.33 (0.184)

*MD* mean difference, *I*^2^ *(p)* heterogeneity and *p*-value, *Z (p)* test for overall effect and *p*-value

*BMI* body mass index, *BMD* bone mineral density

*Statistical significance: p ≤ 0.05

### Analysis of Sex Differences on Body Composition 12-Months Post-BS

Our sub-analysis by sex showed decreases in all body composition and anthropometric outcomes assessed for boys and girls (Supplementary Table 2). Significant reductions were observed in girls for lean mass (− 10.9 kg; 95% CI; − 13.1; − 8.6; *z* =  − 9.5; *p* < 0.001) and fat mass (− 27.8 kg; 95% CI − 30.4; − 25.2; *z* =  − 21.1; *p* < 0.01), and in boys for fat mass (− 37.9 kg; 95% CI; − 42.8; − 33.0; *z* =  − 15.3; *p* < 0.01). The lack of analysis of lean mass for boys and fat-free mass for both sexes was due to insufficient reporting of these outcomes in the selected studies. Body weight losses after surgery in boys (− 46.6 kg; 95% CI; − 53.3; − 39.9; *z* =  − 13.6; *p* < 0.001) seemed to be higher than in girls (− 37.9 kg; 95% CI; − 44.3; − 31.5; *z* =  − 11.6; *p* < 0.001). BMI reductions were also higher in boys (− 15.9 kg; 95% CI; − 18.3; − 13.4; *z* =  − 13.6; *p* < 0.001) than in girls (− 14.2 kg; 95% CI; − 16.1; − 12.3; *z* =  − 14.8; *p* < 0.001). Notwithstanding, at baseline, average body weight was also higher in boys (140.74 kg ± 19.25) than in girls (133.63 kg ± 20.65).

### Type of Surgery-Dependent Analysis on Body Composition

Both SG and RYGB resulted in significant changes in body composition outcomes, namely in fat mass (SG, − 27.9 kg; 95% CI; − 32.6; − 23.2; *z* =  − 11.6; *p* < 0.001; RYGB: − 31.0 kg; 95% CI; − 33.5; − 28.5; *z* =  − 24.4; *p* < 0.001), lean mass (SG, − 7.8 kg; 95% CI; − 9.9; − 5.6; *z* =  − 7.1; *p* < 0.001; RYGB, − 8.8 kg; 95% CI; − 11.5; − 6.1; *z* =  − 6.4; *p* < 0.001), and fat-free mass (SG, − 6.3 kg; 95% CI; − 7.7; − 4.8; *z* =  − 8.5; *p* < 0.001; RYGB, − 6.9 kg; 95% CI; − 10.8; − 3.0; *z* =  − 3.5; *p* < 0.001). Regarding anthropometric outcomes, body weight (SG, − 37.4 kg; 95% CI; − 40.7; − 34.1; *z* =  − 22.2; *p* < 0.001; RYGB, − 41.4 kg; 95% CI; − 44.6; − 38.2; *z* =  − 25.3; *p* < 0.001), and BMI (SG, − 13.3 kg; 95% CI; − 14.4; − 12.3; *z* =  − 25.5; *p* < 0.001; RYGB, − 14.9 kg; 95% CI; − 15.8; − 13.9; *z* =  − 30.7; *p* < 0.001) also decreased significantly after both surgical procedures. There were insufficient data to analyze bone mass and waist circumference changes according to BS procedure. Nevertheless, all body composition and anthropometric changes to be more pronounced in patients undergoing RYGB (Supplementary Table 3).

### Differences in Body Composition Between the First 2 Years after RYGB

Sub-analyses were performed to compare the changes in body composition during the first- and second-year post-RYGB (Supplementary Table 4). Fat-free mass could not be analyzed since only one study assessed this outcome [59]. Changes are more pronounced in the first year following RYGB for all outcomes assessed, namely body weight (− 40.84 kg; 95% CI; − 44.80; − 36.87; *z* =  − 20.19; *p* > 0.01), BMI (− 14.51 kg; 95% CI; − 15.77; − 13.26; *z* =  − 22.72; *p* > 0.01), fat mass (− 29.74 kg; 95% CI; − 32.76; − 26.73; *z* =  − 19.35; *p* > 0.01), and lean mass (− 8.23 kg; 95% CI; − 11.22; − 5.23; *z* =  − 5.39; *p* > 0.01), while at the second year, stabilization of these outcomes is more likely to happen.

### Sensitivity Analysis

After removing studies one by one, it was not possible to reduce the heterogeneity in lean mass or fat mass in the analysis that comprised all types of surgeries. Sensitivity analysis only substantially decreased *I*^2^ to 0% for body composition outcomes in the analysis by type of surgery. For the overall effect of surgery analysis, removing the study of Dubnov-Raz et al. [39], resulted in a *I*^2^ decrease below 30% for body weight and BMI. This could be justified because 25% of the sample of this study remained in severe obesity 12 months after surgery. Interestingly, removing this same study in the analysis by type of surgery, resulted in 0% heterogeneity. Heterogeneity for waist circumference significantly decreased to 0% after removing the study of Inge et al. [55] which assessed only girls. By removing the study of Nimmala et al. [35], the heterogeneity of lean mass analysis also decreased when SG was performed. This study reported the lowest lean mass losses and the higher sample size among all studies included. Similarly, the removal of Beamish et al. [38] (only girls) for lean mass in RYGB and Dubnov-Raz et al. [39] for fat mass in SG reduced *I*^2^ to 0% (Supplementary Table 4). The removal of these studies did not change the results.

## Discussion

The effect of BS in adults body composition is widely documented, but few studies have assessed these outcomes in children and adolescents. There is some concern of performing this procedure during growth due to the potentially extensive lean and fat-free mass losses, which may have future negative health consequences. Our analysis suggests that BS, similarly to adults, is extremely effective in reducing fat mass, body weight, BMI, and waist circumference in adolescents assessed 12 months after surgery. Despite these benefits, a significant reduction in lean mass and fat-free mass was also observed. Body weight, BMI, and fat mass losses seem to be higher in boys than in girls. Both types of surgery, RYGB and SG, were effective in reducing body fat and obesity related anthropometric outcomes; however, higher losses seem to be observed in patients who underwent RYGB. Finally, in patients submitted to RYGB, body composition reductions were more pronounced in the first-year post-surgery whereas in the second year a stabilization was frequently observed.

Several studies have investigated the effect of BS on body weight and BMI in a younger population. However, few studies have assessed changes in body composition in this population. Beyond the positive reductions observed in body weight, fat mass, BMI, and waist circumference, a reduction of obesity-related comorbidities after surgery is also documented [24], evidencing BS effectiveness during adolescence and young adulthood for improving cardiometabolic health. Despite these benefits, the surgery associated energy deprivation also leads to significant decreases in muscle mass. This might negatively affect whole-body metabolism such as aerobic capacity [62], regulation of resting metabolic rate, and possible lead to long-term musculoskeletal issues [63]. Of note, muscle mass losses in adolescents have been associated with an increased risk of metabolic syndrome [64] and nonalcoholic fatty liver disease development [65]. Noteworthy, in weight loss multidisciplinary intervention programs (MIP), children and adolescents with obesity were able to, not only decrease their body weight and fat mass, but also maintain [66–68] or even increase their lean mass [69] and fat-free mass [70] percentage. In contrast, patients who underwent SG showed a higher muscle mass loss and protein-energy malnutrition than those undergoing MIP [71]. Therefore, the question remains if the drastic energy deprivation and associated gastrointestinal anatomic, physiologic, and endocrine changes associated with bariatric surgery [72], and the consequently pronounced body weight loss during the growth period could lead to negative long-term consequences, especially of the musculoskeletal system.

Our results show that whole-body BMD did not decrease 12 months after BS, contrasting with most of the findings in adults [73]. However, these results must be carefully interpreted, firstly due to the reduced number of studies included in our analysis (only three studies) and secondly, because during adolescence bone mass is expected to increase. Therefore, since adolescents are going through an active bone modeling phase, increases in bone mass should normally be anticipated. Of note, the peak bone mass (PBM), which is considered the highest amount of bone mass accumulated at the end of the growth period, can be reached during the second [74], or even the third decade of life [75, 76]. PBM is an important determinant of bone strength and bone health [77, 78] and the age of higher bone accrual acquisition is approximately 12 years for girls and 14 years for boys [79]. In this regard, a possible negative effect on bone acquisition promoted by bariatric surgery during this phase, such as due to energy deprivation, might impair PBM achievement and consequently increase the risk of age-related bone disorders, such as osteoporosis, and increased fracture risk [80]. Of note, it is important to recognize that severe obesity, by itself, is also considered harmful for bone quality [81]. Therefore, it is possible that performing BS during the period of accelerated growth and development might impair bone health to the same extent as long term exposure to severe obesity. Curiously, one study observed significantly reduced fat-free mass in adolescents 5 years post-RYGB in comparison with non-surgical controls [60]. Although our results suggested no negative effect of BS on bone mass, the question remains whether the growth observed was sufficient for that expected period of age. As a major limitation hindering the possibility to adequality address this question, few studies provide a control group to compare bone accrual during follow-up after BS. For instance, in Misra et al. 2020b [32••], the adjusted BMD increased less in the surgical group in comparison with non-surgical controls, and the BMD *Z*-score also decreased in most studies [32••, 38, 40]. In this context, in order to identify bone growth restrictions, we performed a BMD *Z*-score analysis 12 months after surgery, but no significant differences were found.

Although few studies reported data according to sex, our results are in agreement with most of the current literature available for adults, in which males present higher reductions in fat mass [82] and higher preservation of lean mass [83] when compared to females. Curiously, our study indicates that, in adolescents, despite a higher fat mass loss observed in boys [38, 39, 60], lean mass [38] and fat-free mass [60] tend to be more preserved than in girls. Interestingly, the sensitivity analysis showed that the heterogeneity in lean mass following RYGB decreased after removing the study of Beamish et al. A (only girls) [38] (Supplementary Table 4; Analysis 11). Moreover, both studies providing data by sex, [38, 55] reported higher lean mass losses in girls compared to boys (Beamish et al. B) or when results are presented for both sexes together [60]. This result suggests that RYGB might promote higher lean mass losses in girls than in boys. This higher lean mass preservation in boys might be explained by a favorable hormonal context with higher circulating testosterone levels in boys during adolescence, which might contribute to mitigating the detrimental effect of BS on lean mass [84]. The higher body weight loss observed in boys can also be related to their higher baseline values, providing a greater margin for weight loss compared to girls. Despite these observations, due to the scarcity of studies providing data according to sex, it was not possible to perform a meta-analysis to clarify if the effect of BS in adolescents’ body composition is sex dependent.

Regarding the analysis according to the type of surgery, both procedures analyzed (SG and RYGB) promoted a reduction in all the selected body composition and anthropometric outcomes in adolescents, except for bone mass. Regarding the results heterogeneity, when data was analyzed according to type of surgery and the study of Dubnov-Raz et al. [39] was removed, a heterogeneity of 0% was achieved. This suggests that different surgical procedures may have different effects on body composition outcomes, increasing the heterogeneity in our analysis. In fact, different types of surgery can lead to different long-term effects [46]. Of note, a recent meta-analysis carried out in data from adults showed that, although RYGB promoted higher lean mass losses than LAGB, changes following RYGB and SG were similar [85]. Notwithstanding, it is important to consider that, as a malabsorptive surgery, RYGB could elicit more pronounced negative consequences to the musculoskeletal system [86]. In the present analysis, despite patients who underwent RYGB presenting higher losses for all body composition and anthropometric outcomes, it was not possible to directly compare the two types of surgical procedures (SG and RYGB) and determine which was the most effective. It is also important to consider that differences in patient’s baseline characteristics might have influenced the results heterogeneity.

Finally, to assess the long-term effect of BS, we compared the mean differences between the first and second year after RYGB. As expected, and similarly to what is observed in adults, body weight, BMI, lean, and fat mass decreased significantly during the first post-surgery year [15•]. Some studies have observed that, after 12 months, fat mass continues to decrease, whereas lean mass could be maintained or even increase [56, 59]. Our meta-analysis, however, shows only the preservation of these outcomes in a period beyond 12 months. Only one study achieved a significant decrease in fat mass and fat-free mass after the first 12 months following surgery, which could be related to the higher dropout observed in this study [13]. The only study with a follow-up of 5 years after RYGB found no significant regain in body weight or fat mass [60]. Despite no fat-free mass regains being observed 5 years post-surgery, protein supplementation preserved fat-free mass better than in patients who did not follow this nutritional recommendation (− 6.5 ± 4.4 kg versus − 10.5 ± 5.4; *p* = 0.01). This highlights the need to better understand the long-term implication of BS on body composition and to develop adequate countermeasures, such as physical exercise programs and nutritional supplementation, to tackle possible future negative effects.

The high heterogeneity in our overall analysis, possibly caused by the type of surgery or sex-related differences in the response to BS, led to some inconsistencies in our results which rated the changes in body composition outcomes with a “low certainty of evidence” status. For an accurate analysis to determine the effect of different types of surgery or how body composition outcomes may vary according to sex, future studies should provide separate data by sex and surgical procedure.

The major strength of our review is to be the first one to summarize the available evidence on the effect of BS on adolescents’ body composition and to characterize these changes according to type of surgical procedure and adolescent’s sex. As a major limitation, we identified only a reduced number of studies assessing body composition changes in adolescents and no studies assessing these outcomes in children. Moreover, some reports included in our analysis were derived from the same study and analyzed the same sample, which may have contributed to bias our results.

## Conclusion

Bariatric surgery is an effective option, when clinically indicated, to treat adolescents with severe obesity leading to significant and lasting reductions in body weight, BMI, and fat mass. Significant reductions in lean mass and fat-free mass were also observed and deserve more investigation, particularly in studies with longer follow-ups. In general, boys showed higher reductions in body weight, BMI, and fat mass than girls. Moreover, sensitivity analysis also showed that in patients who underwent RYGB, the heterogeneity in lean mass only decreased after removing studies that enrolled only girls. This finding could suggest sex-related differences in the response to BS. Patients who underwent RYGB seem to show higher body weight, BMI, and fat mass losses than those submitted to SG; however, the lack of studies comparing both types of surgery hinders an effective comparison between them. Future network meta-analysis making a direct comparison between SG and RYGB could address this limitation. Our results also evidence that changes in body composition are more pronounced during the first 12 months after surgery and that after this, there is a trend for losses attenuation. Lean, fat-free, and bone mass losses are a special concern in adolescents since they are undergoing an important musculoskeletal developmental phase and few studies have assessed these outcomes. Thus, more longitudinal studies including a control group are necessary to adequately address the question regarding the possible negative long-term effect of BS, especially on bone development. Beyond that, more investigation should be encouraged to compare sex-related differences in the response to BS and the effect of different types of BS procedures on adolescents’ body composition.

### Supplementary Information

Below is the link to the electronic supplementary material.

Supplementary file1 (DOCX 352 KB)
