# Supplementary material for: Body Composition Changes in Adolescents Who Underwent Bariatric Surgery: A Systematic Review and Meta-analysis
Source: Curr Obes Rep. 2024 Jan 3;13(1):107–20. doi: 10.1007/s13679-023-00549-6 (PMC10933211; doi:10.1007/s13679-023-00549-6)
Supplement: Supplementary file 1 — Supplementary file1 (DOCX 352 KB) [file 13679_2023_549_MOESM1_ESM.docx]

**Body composition changes in adolescents who underwent bariatric surgery: A systematic review and Meta-analysis.**

Andréa Bezerra^1,2^, Giorjines Boppre^1,2,3^, Laura Freitas^1,2^, Francesca Battista^4,*^, Federica Duregon^4^, Sara Faggian^4^, Luca Busetto^5^, Andrea Ermolao^4,6^, Hélder Fonseca^1,2^.

^1^Research Centre in Physical Activity, Health and Leisure (CIAFEL), Faculty of Sport, University of Porto, Porto, Portugal.

^2^ Laboratory for Integrative and Translational Research in Population Health (ITR), Porto, Portugal.

^3^ Human Motricity Research Center, University Adventista, Chillean, Chile.

^4^ Sports and Exercise Medicine Division, Department of Medicine, University of Padova, Padova, Italy.

^5^ Department of Medicine, University of Padova, Italy.

^6^ Clinical Network of Sports and Exercise Medicine of the Veneto Region, Padova, Italy.

*Corresponding author: [francesca.battista@unipd.it](mailto:francesca.battista@unipd.it)

Table of content

[Supplementary file 2](#_Toc136638728)

[Risk of bias- Fig 1 2](#_Toc136638729)

[GRADE- Certainty of evidence 3](#_Toc136638730)

[Meta-analysis- Table 2 4](#_Toc136638731)

[Meta-analysis- Table 3 5](#_Toc136638732)

[Meta-analysis- Table 4 6](#_Toc136638733)

[Meta-analysis- Table 5 7](#_Toc136638734)

# Supplementary file

##
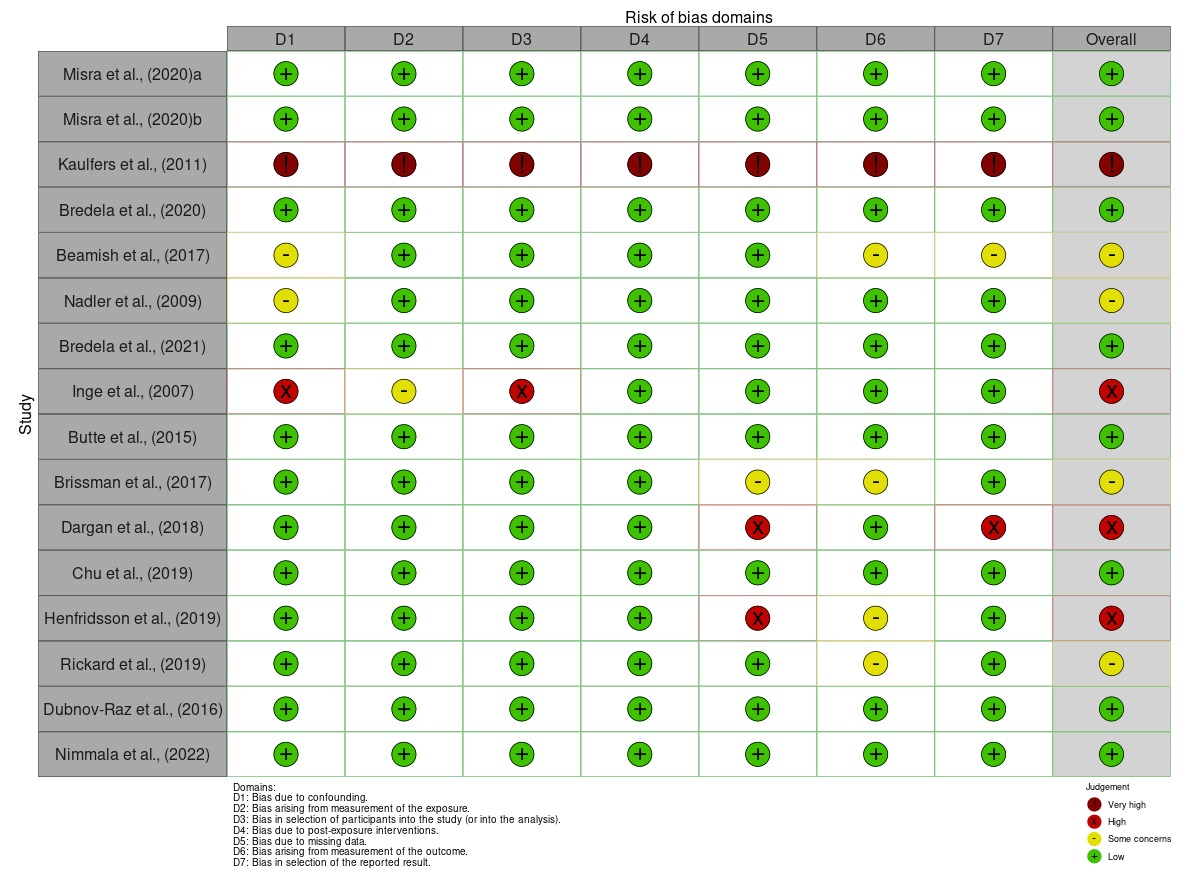
Risk of bias- Fig 1


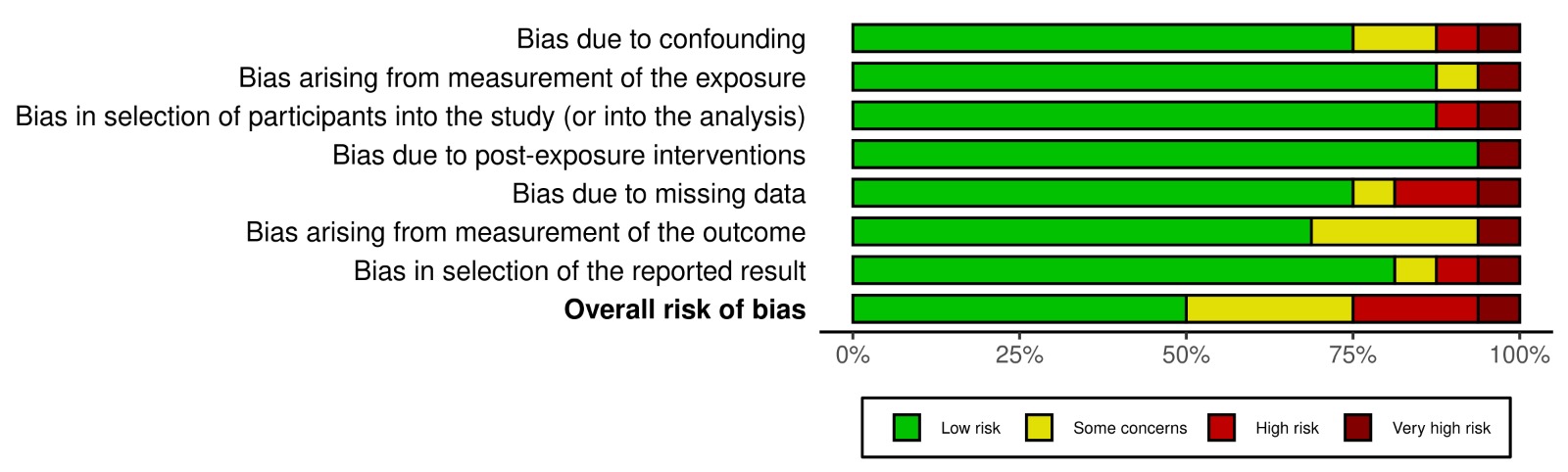


**Figure 1**: Risk of bias assessment of included studies illustrated by traffic light and risk-of- bias summary plot.

## GRADE- Certainty of evidence

**Table 1**: GRADE analysis

| **Outcomes** | **Certainty assessment** | | | | | | | **№ of patients** | | **Effect** | **Certainty** |
| --- | --- | --- | --- | --- | --- | --- | --- | --- | --- | --- | --- |
| **Body composition** | **№ of studies** | **Study design** | **Risk of bias** | **Inconsistency** | **Indirectness** | **Imprecision** | **Other considerations** | **[intervention]** | **[comparison]** | **Absolute (95% CI)** |  |
| Lean Mass | 9 | observational studies | Serious ^a^ | Serious ^b^ | not serious | not serious | publication bias strongly suspected ^c^ | 277 | 281 | **SMD - 0.94** (-1.21 to -0.68) | ⨁◯◯◯ Very low |
| Fat Mass | 15 | observational studies | Serious ^d^ | very serious ^e^ | not serious | not serious | publication bias strongly suspected ^f^ | 385/385 (100.0%) | 400/400 (100.0%) | **SMD -2.23** (-3.00 to -1.47) | ⨁◯◯◯ Very low |
| Fat- free Mass | 6 | observational studies | not serious | not serious | not serious | not serious | publication bias strongly suspected ^g^ | 108/108 (100.0%) | 119/119 (100.0%) | **SMD -0.71** (-0.92 to -0.50) | ⨁◯◯◯ Very low |

**Explanations:**

a. Four studies were rated as having some concerns and 2 studies as having high risk of bias assessed by ROBINS-E

b. There was observed a presence of moderated I^2^ = 56%

c. There was included different types of bariatric surgery such as RYGB, Sleeve Gastrectomy, and Laparoscopic Gastric Banding and both sex.

d. Five studies were rated as having some concerns and 2 studies as having high risk of bias assessed by ROBINS-E

e. There was observed a presence of substantial I^2^ = 84%

f. There was included different types of bariatric surgery such as RYGB, Sleeve Gastrectomy, and Laparoscopic Gastric Banding and both sex.

g. There was included different types of bariatric surgery such as RYGB, Sleeve Gastrectomy, and Laparoscopic Gastric Banding and both sex.

## Meta-analysis- Table 2

**Table 2:** Effects of bariatric surgery on anthropometry and body composition according to sex

.

| **Outcomes** | **Boys** | | | | **Girls** | | | |
| --- | --- | --- | --- | --- | --- | --- | --- | --- |
|  | k | MD (95% CI) | I^2^ | Z (p) | k | MD (95% CI) | I^2^ | Z (p) |
| **Anthropometry** |  |  |  |  |  |  |  |  |
| Body weight (Kg) | 2 | -46.6 (-53.3; -39.9) | 0% | -13.6 (<0.001) | 4 | -37.9 (-44.3; -31.5) | 58% | -11.6 (<0.001) |
| BMI (Kg.m^-2^) | 2 | -15.9 (-18.3; -13.4) | 14% | -12.8 (<0.001) | 4 | -14.2 (-16.1; -12.3) | 42% | -14.8 (<0.001) |
| **Body composition** |  |  |  |  |  |  |  |  |
| Lean mass (kg) |  | na |  |  | 3 | -10.9 (-13.1; -8.6) | 0% | -9.5 (<0.001) |
| Fat mass (kg) | 2 | -37.9 (-42.8; -33.0) | 0% | -15.3 (<0.01) | 4 | -27.8 (-30.4; -25.2) | 0% | -21.1 (<0.001) |
| Note: k: number of studies; MD: mean difference; I^2^: heterogeneity; Z (p): test for overall effect and p-value.  Abbreviations: BMI: body mass index; na: not available  *Statistical significance: p≤0.05. | | | | | | | | |

## Meta-analysis- Table 3

**Table 3:** Effect of different types of bariatric surgery in anthropometry and body composition

| **Outcomes** | **Sleeve gastrectomy** | | | | **RYGB** | | | |
| --- | --- | --- | --- | --- | --- | --- | --- | --- |
|  | k | MD (95% CI) | I^2^ | Z (p) | k | MD (95% CI) | I^2^ | Z (p) |
| **Anthropometry** |  |  |  |  |  |  |  |  |
| Body weight (kg) | 10 | -37.4 (-40.7; -34.1) | 49% | -22.2 (<0.001) | 6 | -41.4 (-44.6; -38.2) | 0% | -25.3 (<0.001) |
| BMI (kg.m^-2^) | 10 | -13.3 (-14.4; -12.3) | 34% | -25.5 (<0.001) | 6 | -14.9 (-15.8; -13.9) | 0% | -30.7 (<0.001) |
| **Body composition** |  |  |  |  |  |  |  |  |
| Lean mass (kg) | 4 | -7.8 (-9.9; -5.6) | 59% | -7.1 (<0.001) | 4 | -8.8 (-11.5; -6.1) | 44% | -6.4 (<0.001) |
| Fat mass (kg) | 7 | -27.9 (-32.6; -23.2) | 85% | -11.6 (<0.001) | 6 | -31.0 (-33.5; -28.5) | 22% | -24.4 (<0.001) |
| Fat-free mass (kg) | 3 | -6.3 (-7.7; -4.8) | 0% | -8.5 (<0.001) | 2 | -6.9 (-10.8; -3.0) | 0% | -3.5 (<0.0005) |
| Note: k: number of studies; MD: mean difference; I^2^: heterogeneity; Z (p): test for overall effect and p-value.  Abbreviations: BMI: body mass index; RYGB: Roux-n-Y gastric banding.  *Statistical significance: p≤0.05. | | | | | | | | |

## Meta-analysis- Table 4

**Table 4:** Differences in body composition changes between 0-12- and 12-24-months following Bariatric surgery.

| **Outcomes** | **0-12 *vs* 12-24 Months** | | | |
| --- | --- | --- | --- | --- |
|  | k | MD (95% CI) | I^2^ | Z (p) |
| **Anthropometry** |  |  |  |  |
| Body weight (kg) | 4 | -40.84 (-44.8; -36.87) | 0% | -20.19 (<0.01) |
| BMI (kg.m^-2^) | 4 | -14.51 (-15.77; -13.26) | 0% | -22.72 (<0.01) |
| **Body composition** |  |  |  |  |
| Lean mass (kg) | 3 | -8.23 (-11.22; -5.23) | 47% | -5.39 (<0.01) |
| Fat mass (kg) | 4 | -29.74 (-32.76; -26.73) | 8% | -19.35 (<0.01) |
| Note: k: number of studies; MD: mean difference; I^2^: heterogeneity; Z (p): test for overall effect and p-value.  Abbreviations: BMI: body mass index.  *Statistical significance: p≤0.05. | | | | |

##

## Meta-analysis- Table 5

**Table 5:** Sensitivity analysis

| **Outcomes** | **Overall procedures** | | | | | | |
| --- | --- | --- | --- | --- | --- | --- | --- |
|  | k | MD (95% CI) | I^2^ | | | | Z **(p)** |
|  |  |  | Before | | After | |  |
| **Body weight** |  |  |  | | |  |  |
| Analysis 1 | 15 | -38.1 (-40.3; -35.9) | 39% | | | 9.8% | -34.2 **(<0.0001)** |
| **BMI** |  |  |  | | |  |  |
| Analysis 2 | 15 | -13.7 (-14.5; -12.9) | 37% | | | 29.6% | -32.9 **(<0.0001)** |
| **Waist circumference** |  |  |  | | |  |  |
| Analysis 3 | 2 | -22.6 (-29.2; -16.3) | 73% | | | 0% | -6.9 **(<0.0001)** |
| **Lean mass** |  |  |  | | |  |  |
| Analysis 4^‡^ |  |  |  | | |  |  |
| **Fat mass** |  |  |  | | |  |  |
| Analysis 5^‡^ |  |  |  | | |  |  |
|  |  | **Sex** | | | | | |
| **Body weight (girls)** |  |  | | | | | |
| Analysis 6^‡^ |  |  | |  | |  |  |
| **BMI (girls)** |  |  | |  | |  |  |
| Analysis 7 | 3 | -13.6 (-14.9; -12.3) | | 42% | | 0% | -21 **(<0.001)** |
|  |  | **Type of surgery** | | | | | |
| **Body weight (SG)** |  |  | |  | |  |  |
| Analysis 8 | 7 | -35.8 (-38.5;-33.1) | | 49% | | 0% | -26 **(<0.001)** |
| **BMI (SG)** |  |  | |  | |  |  |
| Analysis 9 | 7 | -12.6(-13.5; -11.6) | | 34% | | 0% | -26.6 **(<0.001)** |
| **Lean mass (SG)** |  |  | |  | |  |  |
| Analysis 10 | 3 | -8.9 (-10.7;-7.1) | | 59% | | 0% | -9.5 **(<0.001)** |
| **Lean mass (RYGB)** |  |  | |  | |  |  |
| Analysis 11 | 3 | -7.2 (-9.8; -4.6) | | 44% | | 0% | -5.5 **(<0.001)** |
| **Fat mass (SG)** |  |  | |  | |  |  |
| Analysis 12 | 4 | -25.3 (-26.1; -24.5) | | 85% | | 0% | -63 **(<0.001)** |
| Note: k: number of studies included in the analysis, MD: mean difference; 95% CI: confidence interval, I^2^: heterogeneity, Z (p): test for overall effect and p-value.  Abbreviations: BMI: body mass index; SG: sleeve gastrectomy; RYGB: Roux-n-y gastric bypass  For analysis 1, 2, 8, 9 and 12: The study of Dubnov et al. was removed.  For analysis 3 and 7: The study of Inge et al. was removed.  For analysis 10: The study of Nimmala et al. was removed.  For analysis 11: The study of Beamish et al. A (only girls) was removed.  For analysis 4, 5, and 6: Despite removing studies one by one, the heterogeneity did not decrease bellow 30%.  *Statistical significance: p≤0.05. | | | | | | | |
